# Postpartum State, but Not Maternal Caregiving or Level of Anxiety, Increases Medial Prefrontal Cortex GAD_65_ and vGAT in Female Rats

**DOI:** 10.3389/fgwh.2021.746518

**Published:** 2022-02-08

**Authors:** Christina M. Ragan, Eman I. Ahmed, Erika M. Vitale, Katrina Linning-Duffy, Stephanie M. Miller-Smith, Jamie Maguire, Joseph S. Lonstein

**Affiliations:** ^1^Neuroscience Program, Michigan State University, East Lansing, MI, United States; ^2^School of Biology and Undergraduate Neuroscience Program, Georgia Institute of Technology, Atlanta, GA, United States; ^3^Behavioral Neuroscience Program, Department of Psychology, Michigan State University, East Lansing, MI, United States; ^4^Department of Neuroscience, Tufts University School of Medicine, Boston, MA, United States

**Keywords:** anxiety, GABA, lactation, maternal behavior, medial prefrontal cortex, reproductive state

## Abstract

Upregulation of the inhibitory neurotransmitter, GABA, is involved in many of the behavioral differences between postpartum and nulliparous female rodents. This is evidenced by studies showing that pharmacological blockade of GABAergic activity impairs maternal caregiving and postpartum affective behaviors. However, the influence of motherhood on the capacity for GABA synthesis or release in the medial prefrontal cortex (mPFC; brain region involved in many social and affective behaviors) is not well-understood. Western blotting was used to compare postpartum and nulliparous rats in protein levels of the 65-kD isoform of glutamic acid decarboxylase (GAD_65;_ synthesizes most GABA released from terminals) and vesicular GABA transporter (vGAT; accumulates GABA into synaptic vesicles for release) in the mPFC. We found that postpartum mothers had higher GAD_65_ and vGAT compared to virgins, but such differences were not found between maternally sensitized and non-sensitized virgins, indicating that reproduction rather than just the display of maternal caregiving is required. To test whether GAD_65_ and vGAT levels in the mPFC were more specifically related to anxiety-related behavior within postpartum mothers, we selected 8 low-anxiety and 8 high-anxiety dams based on their time spent in the open arms of an elevated plus maze on postpartum day 7. There were no significant differences between the anxiety groups in either GAD_65_ or vGAT levels. These data further indicate that frontal cortical GABA is affected by female reproduction and more likely contributes to differences in the display of socioemotional behaviors across, but not within, female reproductive state.

## Introduction

The early postpartum period is associated with a number of salient behavioral modifications including elevated interest in neonates, the performance of caregiving activities, and a suppression of anxiety- and fear-related behaviors that facilitates mothers' positive focus on the offspring [for reviews see (1, 2)]. The neurochemical underpinnings of these postpartum behaviors include numerous steroids, neuropeptides, and classical neurotransmitters (1, 3, 4). Of the latter, a role for the inhibitory neurotransmitter GABA has received considerable attention. For instance, postpartum rats have higher basal GABA release and turnover in a number of brain areas regulating postpartum behaviors (5, 6). Furthermore, the postpartum GABA system is acutely affected by interactions with offspring, such that cerebrospinal fluid concentrations of GABA are high when mother rats interact with pups, but drop to almost non-detectable levels within hours after the litter is removed (7).

Such changes in GABAergic activity are functionally relevant for mothers' behavior. Peripheral administration of GABA_A_ receptor (GABA_A_R) agonists or antagonists interfere with most postpartum behaviors, including dams' maternal caregiving and their characteristically low anxiety-related behavior (8–14). Furthermore, experiments targeting specific forebrain sites have identified the mPFC as a locus for GABA's involvement in both maternal caregiving and postpartum affective behaviors (8, 14, 15).

Studies exploring differences between parous and nulliparous female laboratory rodents in their central GABA_A_ or benzodiazepine receptor binding have yielded negative results (16–19). This suggests that it would be more fruitful to delve into the presynaptic mechanisms underlying changes across female reproduction in central GABA synthesis and release, including glutamate decarboxylase (GAD_65_) and the vesicular GABA transporter (vGAT), which we focus on in the present experiments.

Glutamate is decarboxylated into GABA either by GAD_65_ or the larger GAD_67_ isoform, but unlike GAD_67_ which is found throughout the cytosol and generates the basal GABA necessary for cellular metabolic function, GAD_65_ is predominantly found in neuronal terminals where it synthesizes most of the GABA packaged into synaptic vesicles (20–23). Therefore, GAD_65_ is the primary form of GAD necessary for the GABA that is involved in phasic synaptic inhibition (24–26) as well as for the tonic extrasynaptic inhibition occurring from ambient GABA in the extracellular space (27–29). GAD expression is already known to be affected by reproductive state and maternal interactions with pups in at least three forebrain sites—the olfactory bulb, bed nucleus of the accessory olfactory tract, and rostral lateral septum (30–32)—but other sites implicated in postpartum behaviors have not been examined. The other GABA system protein of interest in the present study is vGAT, which is tightly coupled to GAD_65_ and packages newly synthesized GABA into vesicles for its release at the synapse (23, 33). Although the importance of vGAT for synaptic GABA release is evident by the reduction in inhibitory postsynaptic currents (IPSCs) in brain tissue from vGAT knockout mice (34), the influence of motherhood on vGAT anywhere in the brain is unknown.

An important consideration when investigating neurochemical changes across female reproduction is that nulliparous and parous females not only differ in their endocrine experiences, but also in their behavioral experience. To help distinguish between the neurobiology of female reproductive state and female postpartum behavior, some studies utilize the maternal virgin (or maternally sensitized) model of caregiving (35). Most nulliparous female laboratory rats are not spontaneously maternal but instead find pups aversive. These females can be induced to show maternal behavior, though, by repeatedly exposing them to young pups. Sensitized maternal behavior does not require hormones, and is similar to postpartum caregiving in that it includes nest building, retrieval and licking the pups, nursing-like postures over the litter, and reduced fear and anxiety-like behaviors (8, 36–40). Comparing maternally sensitized virgins to non-sensitized nulliparous females allows insight into the neural basis of maternal socioemotional behaviors without the involvement of pregnancy, parturition, and lactation.

In our first experiment, we compared GAD_65_ and vGAT levels in the mPFC of postpartum day 7 and virgin females. In a second experiment, we then used the maternal sensitization model to examine levels of GAD_65_ and vGAT in a group of ovariectomized, nulliparous rats permitted 1 week of maternal experience and in a group of unexposed nulliparous controls. Lastly, to determine whether the capacity for GABA synthesis and release in the mPFC is associated with individual differences in anxiety-related behaviors within postpartum females (41), we measured GAD_65_ and vGAT in postpartum females that were selected for their relatively high-anxiety or relatively low-anxiety behavior. The results of the three experiments collectively suggest that female reproductive state, but not maternal caregiving alone or degree of postpartum anxiety, is associated with levels of GAD_65_ and vGAT in the mPFC.

## Methods

### Subjects

Adult female Long-Evans rats born and raised in our colony were maintained under a 12:12 h light/dark cycle, constant temperature and humidity, and food and water *ad libitum* as described previously (41). Beginning at 70 days of age, estrous cycles of females intended for the postpartum groups were monitored using a vaginal impedance meter (Fine Science Tools, Foster City, CA), and on a day of proestrus these females were placed in the cage of a sexually experienced Long-Evans male rat for 48 hr. Females were then rehoused with 1 or 2 other mated females for the next 16 days, then singly housed until sacrifice 7 days after parturition (*n* = 15). Litters were culled to mixed-sex groups of 8 pups within 24 h after parturition. Another group of virgin females were not mated, but vaginally smeared daily and sacrificed on a day of diestrus (*n* = 15). Diestrus rats were chosen to compare to the day 7 postpartum rats because these groups are behaviorally very different, but are both in a state of diestrus involving low circulating estradiol and moderate levels of circulating progesterone (42–44). Comparing these groups allowed us to infer that any differences in GAD_65_ or vGAT protein levels would probably not be due to females' ovarian state at sacrifice, but instead other differences between groups in their physiology and behavior.

We further teased apart the behavioral and endocrine influences on central GAD_65_ and vGAT levels by comparing maternally sensitized virgin rats (*n* = 12) to a group of non-sensitized virgin controls (*n* = 11). All subjects were anesthetized with ketamine and xylazine and ovariectomized to ensure that ovarian hormones could not be responsible for differences between groups. Beginning a week after surgery, and using methods similar to our previous studies (36, 45), the to-be-sensitized subjects were exposed in their home cage to three freshly fed 1–7 day old male and female pups obtained from surrogate dams in our colony. Pups were placed in the corner of the cages opposite the subjects' nests. For the next 15 min, females' pup-directed behaviors (sniffing or retrieving each of the three pups, licking the pups, huddling over the pups) were scored by an observer as present or not present. Subjects reached “full maternal behavior” by retrieving all three pups to a single location, licking them, and huddling over them on 2 consecutive test days (46). Each morning the foster pups from the previous day were removed from the subjects' cages, and it was recorded if the pups were warm, to help confirm subjects' maternal or non-maternal state. Those pups were placed back with surrogate dams for at least 2 days to ensure they were well fed before being used again. To better be able to compare the sensitized females in this experiment to the day 7 postpartum females studied in the first experiment, the sensitized females showing full maternal behavior were given an additional 5 days of experience with pups to reach 7 full days of caregiving. This was achieved by continuing to place three freshly-fed pups in the sensitized females' cages each day and observing the subjects for 15 min daily to verify the continuance of their full maternal behavior. The control females never received pups in their cages.

In the final experiment, 40 females from our colony were screened for anxiety-related behavior in an elevated plus maze on postpartum day 7 [see (41) for details]. Briefly, time spent in and frequency of entries into the open and closed arms of an elevated plus maze were recorded during 10-min tests. Subjects were immediately sacrificed after their behavioral test and the 8 highest-anxiety and 8 lowest-anxiety females based on their percentage of time spent in the open arms of the plus maze were selected for brain analysis. All procedures were conducted in compliance with the Institutional Animal Care and Use Committee (IACUC) at Michigan State University.

### Tissue Preparation

**Figure 1:**
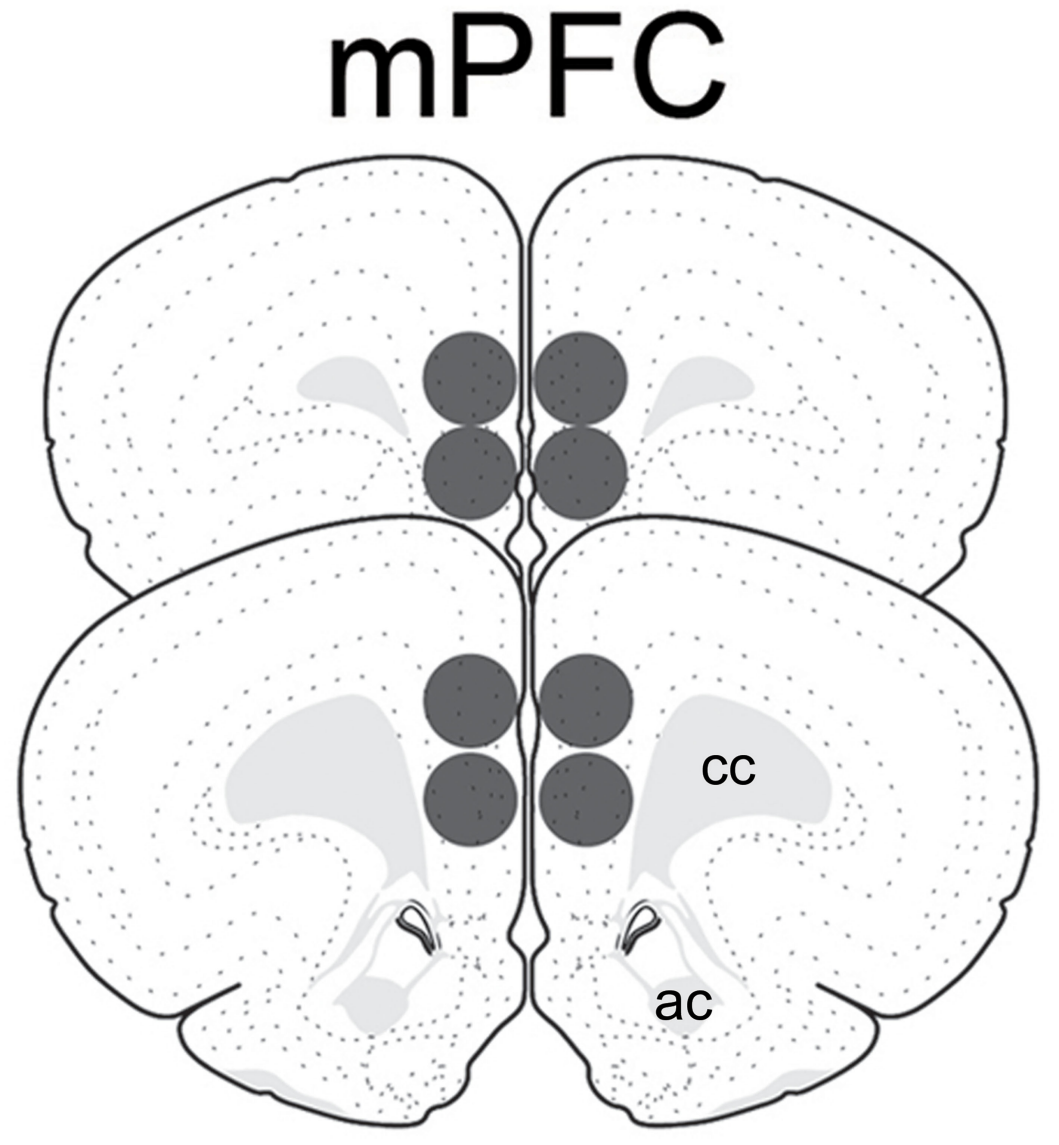
Schematic representation of tissue punches within the medial prefrontal cortex analyzed for GAD_65_ and vGAT. Images modified from Swanson (47). ac, anterior commissure; cc, corpus callosum.

Animals were rendered unconscious by brief exposure to CO_2_ and decapitated. Brains were removed, frozen with ice cold isopentane, and stored at −80°C until further processing. Brains were later coronally sectioned with a cryostat (LEICA CM 1950, Nussloch, Germany) into 500 μm-thick sections containing the medial prefrontal cortex (mPFC; ~ +3.2 to 2.2 mm from bregma, plates 8–10 from Swanson's atlas) (47). Tissue punches were made bilaterally using a 1.0 mm-diameter stainless steel brain punch (Stoelting CO # 57397, Wood Dale, IL) and included the prelimbic and infralimbic subregions (Figure 1). The punches were placed in centrifuge tubes containing 50 μl of RIPA buffer that consisted of lysis buffer, protease inhibitor cocktail, sodium orthovanadate, and 10 mg/ml PMSF reagent (Santa Cruz SC-24948, Santa Cruz, CA). Punches were homogenized using a sonic dismembrator (Fisher Scientific, Pittsburgh, PA), centrifuged for 15 min at 4°C at 15,000 rpm, and the supernatants collected. Samples were frozen and stored at −80°C until Western blotting.

### Western Blotting

Protein concentrations were determined using BCA protein assay kits (Thermo Scientific Pro #23227 Rockford, IL) and a microplate reader (Bio-Rad iMark, Hercules, CA). Samples were immunoblotted for GAD_65_, vGAT, and then GAPDH as the loading control. Ten micrograms of total protein from each sample were denatured at 95°C for 5 min and gel electrophoresed on 10% Tris-Glycine precast gels (NB10-010; Nusep; Bogart, GA). Each gel contained samples from both groups within each of the three experiments (parous/nulliparous; sensitized/unsensitized; high anxiety/low anxiety). Proteins were transferred to polyvinylidene difluoride membranes using an iBlot system (Invitrogen # iB4010, Grand Island, NY). The membranes were washed three times for 10 min each in TBS-T (TBS containing 0.05% Tween-20) and blocked with 5% non-fat dry milk in TBS-T for 1 h at room temperature to reduce non-specific binding. The membranes were then incubated in a rabbit anti-vGAT primary antiserum (1:1,500; Millipore #AB2257, Temecula, CA) in TBS-T and 0.02% sodium azide overnight at 4°C. Following that incubation, membranes were washed three times for 10 min each in TBS-T, incubated in a peroxidase-conjugated anti-rabbit IgG secondary antiserum (1:5,000; Cell Signaling #7074, St. Louis, MO) for 1 h at room temperature, and rinsed in TBS three times. Detection of immunoreactive bands was accomplished using enhanced Luminol chemiluminescence kits (Santa Cruz SC-2048, Santa Cruz, CA), after which the membranes were exposed to films (Optimum Blue Sensitive film, XR-0810-100, Life Science Products, Frederick, CO) that were developed and fixed (Kodak X-OMAT 1000A, Rochester, NY).

Following immunoblotting for vGAT, the membranes were stripped and reblotted for GAD_65_. Membranes were rinsed twice for 10 min each time in TBS-T and incubated in stripping buffer (Thermo Scientific #46430, Rockford, IL) for 15 min. After stripping and rinsing four times, blotting was performed as described above but with a rabbit anti-GAD_65_ antiserum (1:2,000; Cell Signaling # 5843S) overnight at 4°C followed by incubation in a peroxidase-conjugated anti-rabbit IgG secondary antiserum (1:5,000; Cell Signaling #7074) for 1 hr at room temperature. Immunoreactive bands were again detected using chemiluminescence kits and exposed to film as described above. Membranes were then stripped a second time and reprobed as described above using an overnight incubation at 4°C in a mouse anti-GAPDH antiserum (1:500; Millipore #MAB374, Billerica, MA) followed by a peroxidase-conjugated rabbit anti-mouse secondary antiserum (1:80,000; Sigma-Aldrich #A9044, St. Louis, MO) for 1 h at room temperature. Immunoreactive bands were detected and membranes exposed to film as described above.

### Image and Data Analysis

**Figure 2:**
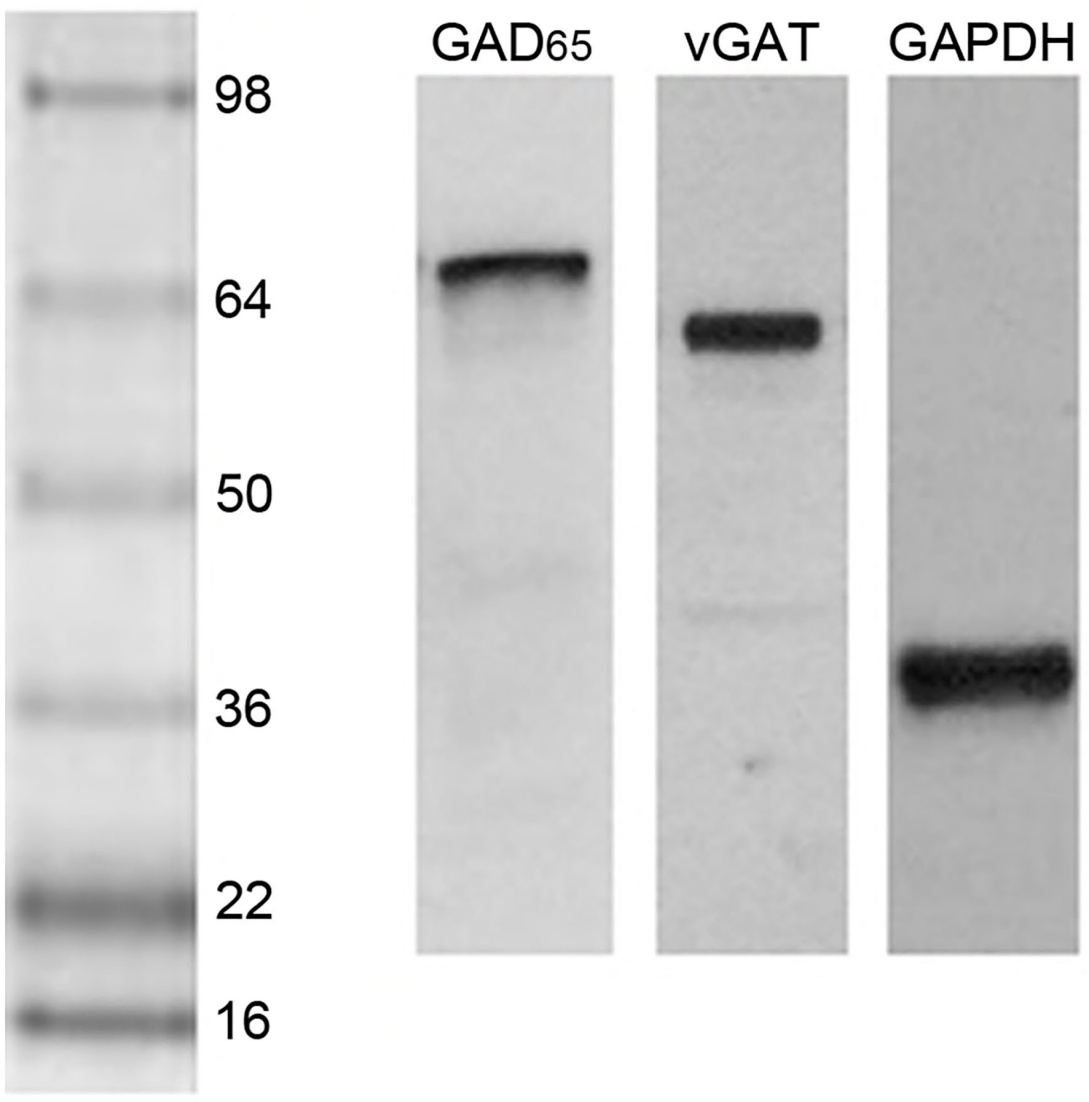
Western blot lanes containing the mPFC of representative postpartum female rats and probed for GAD_65_, vGAT, and GAPDH (left to right). The protein ladder is shown on the left side.

A single, dense immunoreactive band at the expected molecular weight was found after blotting with each antiserum (Figure 2). Films were placed on a light box and digital images captured with a Nikon E400 microscope and camera. Image J (NIH, Bethesda, MD) was used to determine the integrated density of the immunoreactive band for each subject's GAD_65_ and vGAT. As very commonly done with Western blot data, a ratio between the optical density of the bands for each of the two proteins of interest and the optical density of their band for GAPDH was created for each subject (48). Because the GAD_65_ ratio data from the first experiment comparing postpartum dams to nulliparous females failed Levene's test of homogeneity of variance (*p* = 0.015), these data were analyzed with a non-parametric Mann-Whitney *U*-test. The other comparisons were performed with independent *t*-tests. One pup-exposed nulliparous rat in experiment 2 did not reach the criteria for full maternal behavior, so was removed from the study. vGAT was not detected on the blot of one female from the low-anxiety group and she was omitted from the vGAT analyses in experiment 3. Furthermore, GAD_65_ data in the high- and low-anxiety comparison study were not normally-distributed and one outlier from the high-anxiety group was detected *via* a Grubb's test (*p* < 0.05). Data were normally-distributed after this outlier was removed from the analyses. The null hypotheses for all analyses were that the two groups being compared (parous/nulliparous; sensitized/unsensitized; high anxiety/low anxiety) would not differ in their mean vGAT or GAD_65_ levels. Effects sizes for all analyses were calculated with Cohen's *d*. Statistical analyses were conducted using IBM SPSS Version 26 software. Statistical significance was indicated by *p* < 0.05.

## Results

**Figure 3:**
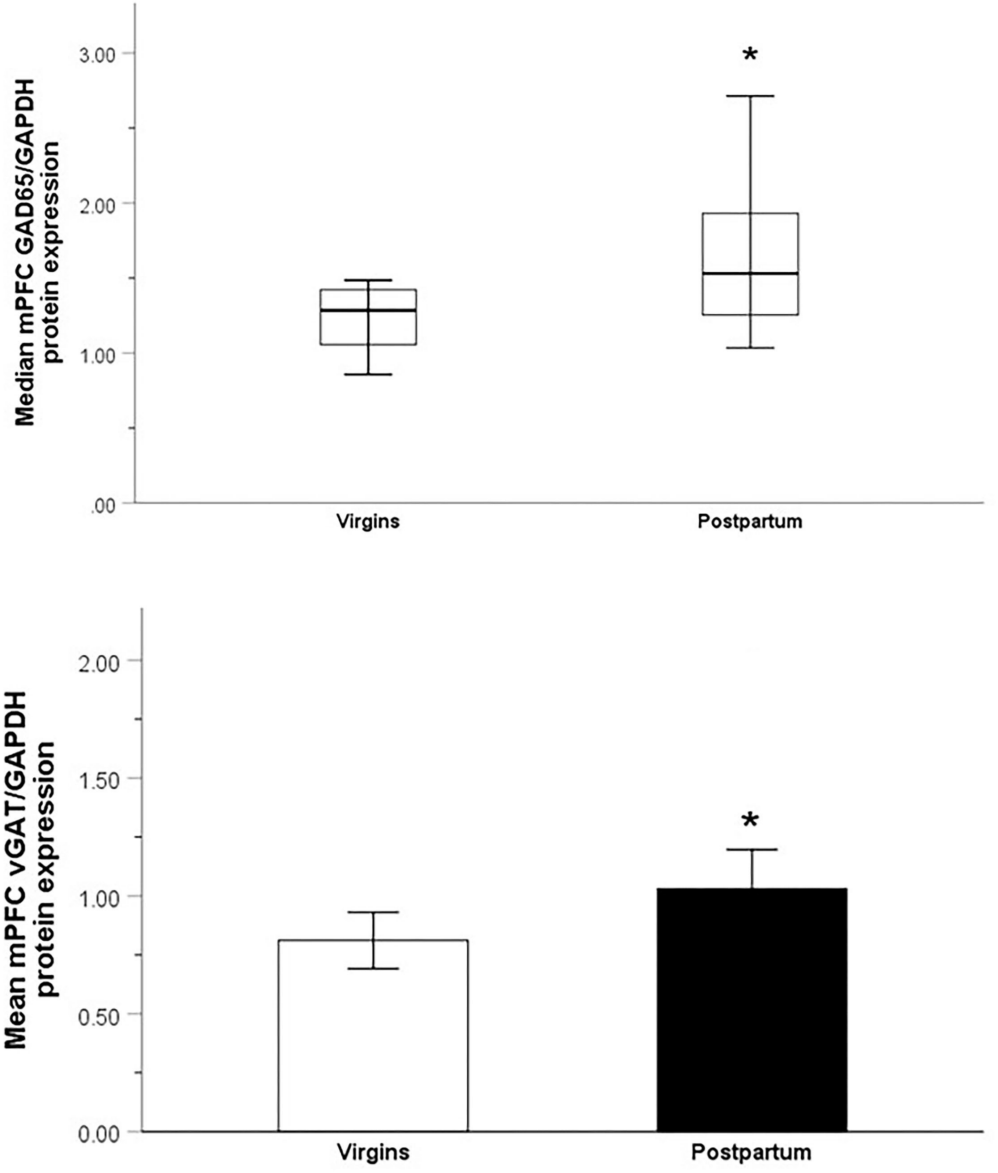
Relative optical density of GAD_65_-immunoreactive bands (top; Median ± IQR) and vGAT-immunoreactive bands (bottom; Mean ± SEM) from the mPFC of nulliparous virgin and postpartum rats. *p < 0.05.

Nulliparous and parous females differed in their median mPFC levels of GAD65 (*Mdn* = 1.28, IQR = 0.38 vs. *Mdn* = 1.53, IQR = 0.78, respectively; *U*_30_ = 54, *p* = 0.02, *d* = 0.99) and mean mPFC levels of vGAT (*M* = 0.81, SE = 0.06 vs. *M* = 1.03, SE = 0.08, respectively; *t*_29_ = 2.15, *p* = 0.04, *d* = 0.79). Parous females had higher levels of both proteins (Figure 3).

The maternally-sensitized nulliparous females took a median of 4 days (mean = 4.8, range = 3–8 days) from the start of pup exposure to show the 2 days of pup responsiveness to reach the criteria for full maternal behavior. They then received an additional 5 days of experience with pups (total 7 days) in order to match the maternal experience of the dams in the first experiment. Despite this extensive experience with pups, sensitized and unexposed control females did not differ in their mean levels of GAD65 [(*M* = 0.16, SE = 0.001 vs. *M* = 0.18, SE = 0.12, respectively; *t*_21_ = 1.11, *p* = 0.28, *d* = 0.46, 95% CI (−0.17, 0.06)], or vGAT [(*M* = 0.55, SE = 0.65 vs. *M* = 0.66, SE = 0.90, respectively; *t*_21_ = 1.10, *p* = 0.28, *d* = 0.46, CI (−0.12, 0.34)], in the mPFC.

As could be expected, the low-anxiety dams from this sample spent 23 ± 3% of their time in open arms of the elevated plus maze whereas the high-anxiety dams spent 6 ± 1% [*t*_13_ = 5.24, *p* < 0.001, *d* = 2.18]. However, the low-anxiety dams and high-anxiety dams did not differ in their mean GAD65 [*M* = 28.17, SE = 2.65 vs. *M* = 24.94, SE = 1.58, respectively; *t*_13_ = 1.01, *p* = 0.33, *d* = 0.52 (CI −3.70, 10.16)], or mean vGAT (*M* = 27.64, SE = 5.72 vs. *M* = 36.89, SE = 8.54, respectively; *t*_13_ = 0.92, *p* = 0.37, *d* = 0.48, (CI −30.95, 12.46)], levels in the mPFC. There were also no significant correlations between the dams' percentage of time spent in the open arms and their levels of GAD_65_ [*r*_15_ = 0.49, *p* = 0.09 (CI −0.001, 0.017)] or vGAT [*r*_15_ = −0.36, *p* = 0.23, (CI −0.005, 0.001)] in the mPFC.

## Discussion

Postpartum and nulliparous female laboratory rodents differ in numerous aspects of their central GABA system. GABA concentrations in cerebrospinal fluid, and GABA release or metabolism in a number of forebrain sites (e.g., mPFC, mPOA, and BST), are higher in postpartum mothers compared to non-mothers and decrease when the offspring are removed (5–7, 49). Furthermore, GAD levels in the olfactory bulb, bed nucleus of the accessory olfactory tract, and lateral septum are higher in postpartum rats compared to nulliparous females (30–32). The first goal of the present experiments was to expand upon these findings by comparing nulliparous and parous rats in their protein levels of GAD_65_ (the isoform found in neuronal terminals and necessary for synthesizing GABA for synaptic release) and vGAT (transports GABA into synaptic vesicles) in the mPFC. We found that postpartum female rats had higher levels of both proteins, suggesting that these mothers have increased capacity for GABA synthesis and release. This is consistent with earlier work showing that basal extracellular GABA and turnover are higher in the mPFC of postpartum rats compared to that of cycling virgins (5, 6).

One consequence of elevated GABA synthesis, packaging and release in the postpartum mPFC may be greater high-intensity phasic synaptic inhibition, which requires high GABA production and release. In contrast, the tonic GABA inhibition mediated by high-affinity extrasynaptic GABA_A_Rs occurs under relatively low ambient GABA concentrations (50). Elevated GABA-mediated phasic inhibition in the postpartum mPFC could be involved in a number of behavioral or physiological changes occurring at this time of female reproduction. Related to behavior, the mPFC has widespread cortical and subcortical connections (51, 52) through which it evaluates an array of internal and external signals to bias the processing of competing neural inputs; it thus act as a higher-order, experience-informed regulator of behavioral responding (53, 54). This includes the execution or inhibition of goal-directed and affective behaviors, such as those saliently modified during the postpartum period. In support of a role in caregiving behaviors, the postpartum rat mPFC responds electroencephalographically to nest odors (55) and electrophysiologically to snout contact with offspring (56). Lesioning or chemically inactivating the postpartum mPFC impairs pup retrieval and other maternal behaviors (15, 57, 58). Most GABAergic neurons in the mPFC are interneurons modulating the activity of projection cells (59). However, there must be limits to any benefit of mPFC disinhibition for maternal behaviors because: (1) as indicated just above, additional postpartum inhibition of the mPFC by GABA_A_R agonism or anesthetic-based deactivation impairs pup-directed behaviors (15, 58) and (2) high impulsivity that can result from low mPFC top-down control is associated with poor mothering in female rats (60).

Interpreting the functional significance of our first set of findings comparing across female reproductive state is complicated by the fact that parous females are not only behaviorally different from nulliparae but also differ from them physiologically. For instance, the maternal brain must coordinate numerous physiological adaptations including blunting of the HPA axis, the neuroendocrinological control of milk production and milk letdown, and changes in energy balance (61–65). To help us understand if the changes in GAD_65_ and vGAT in the first experiment were more due to behavioral or physiological adaptations, we then compared groups of virgin ovariectomized female rats that received a maternal sensitization procedure, and then allowed up to a week of caregiving experience with pups to match the experience of the postpartum dams in the first experiment. The sensitized and non-sensitized females did not differ in their mPFC GAD_65_ or vGAT levels. This indicates that the mPFC differences in the first study between parous and nulliparous females were more likely related to postpartum physiological adaptations, although it is also possible that the natural and sensitized models of maternal behavior produce different changes in the GABA system or even utilize different neurochemical systems to converge upon the same behavioral outcome. It is also the case that maternally sensitized virgins show somewhat inferior maternal caregiving behaviors compared to postpartum rats (8, 36–38, 40, 66, 67). The more robust and complete display of the behaviors characteristic of postpartum mothers may require the changes in GAD_65_ and vGAT revealed in the first study. Experiments determining the effects of promoting or inhibiting mPFC GABA signaling on maternal behaviors in sensitized nulliparous rats would help elucidate these possibilities.

It was also possible that our parity differences in mPFC GAD65 and vGAT in the first study were related to parity differences in behaviors other than maternal caregiving. As mentioned above, most early postpartum rats show less anxiety-related behavior when compared to diestrous virgins, a phenomenon requiring that dams have recent physical contact with the litter and is partly mediated by high maternal central GABA_A_R activity (12, 13, 68). Sabihi et al. (14) recently found that the low anxiety state of postpartum rats tested in an EPM was temporarily prevented by GABA_A_R antagonism with bicuculline infused into the mPFC. Conversely, mothers separated from their pups for 4 h before testing showed relatively high anxiety, but not if the GABA_A_R agonist muscimol was infused into their mPFC (14). Muscimol infusion did not further decrease anxiety in the non-separated mothers, indicating that they were already at a ceiling for the effects of mPFC GABA_A_R on postpartum anxiety-related behaviors. Consistent with these results, we here found that within postpartum rats kept with their pups until testing, GAD_67_ and vGAT levels in the mPFC were unrelated to whether dams showed high anxiety or low anxiety behavior in an EPM. Thus, it appears that as long as mothers have reached a particular (i.e., high) threshold of GABAergic activity in their mPFC, exceeding it through natural variation or pharmacological methods may be mostly irrelevant to maternal affective behaviors.

Since maternal caregiving or anxiety state were apparently not alone responsible for differences among parous and nulliparous groups in their GAD_67_ and vGAT expression, differences in their endocrine histories surely contributed instead. Both groups of females were in a diestrous state at sacrifice, but it is notable that serum concentrations of the progesterone metabolite and neurosteroid, allopregnanalone, are significantly higher in postpartum day 8 rats compared to diestrus cycling females (69). Relatively rapid fluctuations in peripheral and central allopregnanalone and other neurosteroids that occur across reproductive states (16, 70) strongly affect central GABA signaling (71–75), but it is yet unknown if the persistent neurosteroid elevations at the end of the first week postpartum are relevant or not to our findings. Assessing GAD_65_ and vGAT after giving dams a 5-α-reductase inhibitor, which prevents progesterone's conversion into its neurosteroid metabolites, would help determine this. Parous females also differ from nulliparous females in the pulses of central and peripheral oxytocin and prolactin release that occur during interactions with pups. These neuropeptides also affect the GABA system and vice-versa (1, 76–78) so could have contributed to the reproductive state differences we found.

It would be valuable for future studies to assess reproductive-state changes in other aspects of the GABA system in the mPFC, as well as elsewhere in the brain. As noted above, studies of female reproductive state differences in GABA_A_ and benzodiazepine receptor binding in numerous brain sites have found mostly negative results (16–19), but reproductive experience does affect the expression of some of the 19 unique receptor subunit proteins that constellate to form GABA_A_Rs (16, 79–81). The subunit makeup of GABA_A_Rs has tremendous consequences for their sensitivity and function (71, 82, 83). In fact, we have found that expression of the delta subunit of the GABA_A_R, which is found extrasynaptically and mediates tonic inhibition under low ambient GABA concentrations, is significantly higher in the midbrain periaqueductal gray [PAG—a site involved in postpartum nursing, anxiety, and aggression (36, 84, 85)] of postpartum rats compared to diestrus virgins (Ahmed et al., unpublished data). To gain an even better picture of how female reproductive state affects the central GABA system, it would also be valuable to measure mPFC levels of the GABA plasma membrane transporter (GAT1), which can remove GABA from the extracellular space as well as increase GABA concentrations *via* a non-vesicular reverse transport mechanism (86).

## Data Availability Statement

The raw data supporting the conclusions of this article will be made available by the authors, without undue reservation.

## Ethics Statement

The animal study was reviewed and approved by Institutional Animal Care and Use Committee (IACUC) at Michigan State University.

## Author Contributions

CR: contributed to running animal and Western blot experiments, analyzing data, creating figures, and manuscript writing. EA: ran Western blot experiments and conducted data analyses. EV: ran animal Western blot experiments and conducted data analyses. KL-D: ran Western blot experiments. SM-S: contributed to the experiments' conceptualization, developed the Western blot protocols, conducted the pilot experiments, and contributed to the manuscript writing. JM: contributed to discussions about analyzing, interpreting the data, and manuscript editing. JL: contributed by conceptualizing the experiments, analyzing data, creating figures, writing, and editing the manuscript. All authors contributed to the article and approved the submitted version.

## Funding

This research was supported in part by NICHD Grant R01HD057962 to JL.

## Conflict of Interest

The authors declare that the research was conducted in the absence of any commercial or financial relationships that could be construed as a potential conflict of interest.

## Publisher's Note

All claims expressed in this article are solely those of the authors and do not necessarily represent those of their affiliated organizations, or those of the publisher, the editors and the reviewers. Any product that may be evaluated in this article, or claim that may be made by its manufacturer, is not guaranteed or endorsed by the publisher.
